# Supplementary material for: Identification and functional analysis of non-coding regulatory small RNA FenSr3 in Bacillus amyloliquefaciens LPB-18
Source: PeerJ. 2023 May 15;11:e15236. doi: 10.7717/peerj.15236 (PMC10194069; doi:10.7717/peerj.15236)
Supplement: Supplemental Information 4 [file peerj-11-15236-s004.zip › KO/CK-vs-T1_map/map05220.html]

KEGG PATHWAY: Chronic myeloid leukemia - Reference pathway


|  |  |
| --- | --- |
| **Chronic myeloid leukemia - Reference pathway** |  |

[
Pathway menu
| Organism menu
| Pathway entry
| Show description
| User data mapping
]

Chronic myeloid leukemia (CML) is a clonal myeloproliferative disorder of a pluripotent stem cell. The natural history of CML has a triphasic clinical course comprising of an initial chronic phase (CP), which is characterized by expansion of functionally normal myeloid cells, followed by an accelerated phase (AP) and finally a more aggressive blast phase (BP), with loss of terminal differentiation capacity. On the cellular level, CML is associated with a specific chromosome abnormality, the t(9; 22) reciprocal translocation that forms the Philadelphia (Ph) chromosome. The Ph chromosome is the result of a molecular rearrangement between the c-ABL proto-oncogene on chromosome 9 and the BCR (breakpoint cluster region) gene on chromosome 22. The BCR/ABL fusion gene encodes p210 BCR/ABL, an oncoprotein, which, unlike the normal p145 c-Abl, has constitutive tyrosine kinase activity and is predominantly localized in the cytoplasm. While fusion of c-ABL and BCR is believed to be the primary cause of the chronic phase of CML, progression to blast crisis requires other molecular changes. Common secondary abnormalities include mutations in TP53, RB, and p16/INK4A, or overexpression of genes such as EVI1. Additional chromosome translocations are also observed,such as t(3;21)(q26;q22), which generates AML1-EVI1.

|  |  |  |
| --- | --- | --- |
| Reference pathway | 184% 150% 122% 100% 82% 67% 55% | 图片下载 |
